# The IL‐18 signaling cascade is implicated in aging, tau pathology and synaptic degeneration

**DOI:** 10.1002/alz70855_103549

**Published:** 2025-12-23

**Authors:** Isabel MacKenzie Sarty, Cynthia Picard, Henrik Zetterberg, Kaj Blennow, John C.S. Breitner, Judes Poirier, PREVENT‐AD Research Group

**Affiliations:** ^1^ Douglas Mental Health University Institute, Montreal, QC, Canada; ^2^ Department of Psychiatry and Neurochemistry, Institute of Neuroscience and Physiology, The Sahlgrenska Academy at the University of Gothenburg, Mölndal, Västra Götalands län, Sweden; ^3^ UK Dementia Research Institute at UCL, London, United Kingdom; ^4^ Clinical Neurochemistry Laboratory, Sahlgrenska University Hospital, Mölndal, Västra Götalands län, Sweden; ^5^ Department of Psychiatry and Neurochemistry, Institute of Neuroscience and Physiology, The Sahlgrenska Academy, University of Gothenburg, Mölndal, Sweden; ^6^ Department of Psychiatry, Faculty of Medicine, McGill University, Montreal, QC, Canada; ^7^ Department of Psychiatry, Faculty of Medicine, McGill University, Montréal, QC, Canada; ^8^ Centre for Studies on Prevention of Alzheimer's Disease (StoP‐AD Centre), Douglas Mental Health University Institute, Montréal, QC, Canada

## Abstract

**Background:**

Emerging evidence suggests a bidirectional role of inflammation in Alzheimer's Disease (AD), possibly protecting against amyloid deposition early but exacerbating later tau‐related injury. The NLRP3 inflammasome, a driver of “inflammaging” (chronic low‐grade inflammation increasing with age), is implicated in AD pathogenesis and progression. We explored a pivotal marker of NLRP3 activation, IL‐18, and its signaling cascade through the AD continuum.

**Method:**

We used longitudinal data from the PREVENT‐AD cohort of at‐risk/pre‐symptomatic AD participants, as well as cross‐sectional data from the ADNI cohort of symptomatic AD and cognitively unimpaired (CU) participants. General and mixed linear models analyzed associations among cerebrospinal fluid (CSF) levels of IL‐18 cascade proteins (OLINK *p*‐E‐A, SomaScan), AD pathology markers (p(181)tau, t‐tau, Aβ42) (ELISA, ECLIA), and markers of synaptic damage (OLINK *p*‐E‐A, mass spectrometry), and cognition (RBANS). Post‐mortem brain tissue samples of autopsy‐confirmed AD and CU individuals were used to analyze cortical IL‐18 cascade proteins and their mRNA in relation to AD pathological deposition of tau and Aβ.

**Result:**

CSF IL‐18 levels significantly increased with age, where CSF IL‐18 levels (β=0.0114, *p* = 0.027) and IL‐18 mRNA (β=0.028. *p* = 0.005) in post‐mortem tissue were longitudinally associated with subject age. IL‐18 signaling proteins were significantly associated with increasing tau pathology, both in the CSF (β=0.00411, *p* = 0.003) and in post‐mortem brain tissue (β=0.278, *p* = 0.035). As well, a naturally occurring genetic variant in the IL‐1 receptor gene cluster was related significantly to tau‐related pathological CSF markers and deposits. Subsequent analyses in the PREVENT‐AD cohort demonstrated a significant association of IL‐18 signaling on cognition (β=2.3203, *p* = 0.049) and synaptic marker levels in the CSF (β=0.8737, *p* <0.001).

**Conclusion:**

The IL‐18 signaling cascade is modulated by age and plays a crucial role in the pre‐symptomatic stage of AD. Contrary to expectation, IL‐18 signaling appears to modulate tau pathology throughout the entire AD continuum, having little or no impact on Aβ pathology. We recently described very strong associations between markers of synaptic degeneration, tau pathology, and cognition (https://doi.org/10.1038/s41380‐024‐02884‐z). The presented results on IL‐18 signaling may help explain the relationship between tau pathology, synaptic damage and ensuing cognitive decline.

